# A Useful Hœmostatic

**Published:** 1855-11

**Authors:** 


A Useful Hœmostatic.—M. Morel, a military pharmacien, has prepared the following fluid, which has often been found highly useful:—Tannic acid 1·20, alum 1, rose-water 40 part.—(*Ibid.*)

Cold Irrigations in Convulsions of Children.—On the strength of three cases in which its employment was attended with remarkable success, M. Larasque strongly recommends to notice the practice of irrigating the head in convulsions, with a continuous stream of water the size of the little finger. He employs about 2 quarts of water.—*Rev. Médicale.* 1855. p. 264. M. Schutzenberger, of Strasburg, also relates two well-marked cases (*Bull de Théra.* T. 48. p. 512,) of hydrocephalus, in which recovery was attributed to repeated affusions performed by a watering-pot.—*London Lancet.*

